# Simultaneous optical and electrical *in vivo* analysis of the enteric nervous system

**DOI:** 10.1038/ncomms11800

**Published:** 2016-06-07

**Authors:** Nikolai Rakhilin, Bradley Barth, Jiahn Choi, Nini L. Muñoz, Subhash Kulkarni, Jason S. Jones, David M. Small, Yu-Ting Cheng, Yingqiu Cao, Colleen LaVinka, Edwin Kan, Xinzhong Dong, Michael Spencer, Pankaj Pasricha, Nozomi Nishimura, Xiling Shen

**Affiliations:** 1 School of Electrical and Computer Engineering, Cornell University, Ithaca, New York 14853, USA; 2 Department of Biomedical Engineering, Cornell University, Ithaca, New York 14853, USA; 3 Department of Biomedical Engineering, Duke University, Durham, North Carolina 27708, USA; 4 Center for Neurogastroenterology, Department of Medicine, Johns Hopkins University, Baltimore, Maryland 21205, USA; 5 Solomon H. Snyder Department of Neuroscience, Department of Neurosurgery, Center for Sensory Biology, Johns Hopkins University School of Medicine, Baltimore, Maryland 21205, USA; 6 Howard Hughes Medical Institute, Chevy Chase, Maryland 20815, USA

## Abstract

The enteric nervous system (ENS) is a major division of the nervous system and vital to the gastrointestinal (GI) tract and its communication with the rest of the body. Unlike the brain and spinal cord, relatively little is known about the ENS in part because of the inability to directly monitor its activity in live animals. Here, we integrate a transparent graphene sensor with a customized abdominal window for simultaneous optical and electrical recording of the ENS *in vivo*. The implanted device captures ENS responses to neurotransmitters, drugs and optogenetic manipulation in real time.

## Abstract (web summary)

The enteric nervous system (ENS) plays a key role in regulating gut motility and homeostasis yet it remains a challenging system to record from. Here, the authors develop a novel abdominal window permitting simultaneous optical and electrical recording of mouse ENS system activity over prolonged time periods.

## Graphical abstract

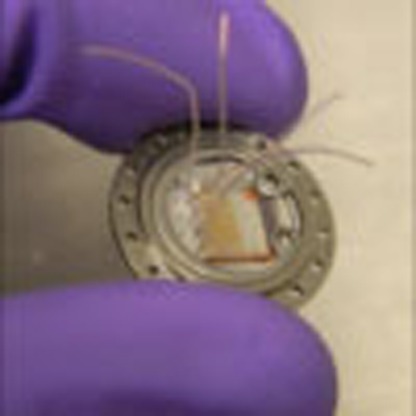

The enteric nervous system (ENS) contains the largest population of neurons in the peripheral nervous system, five times the amount in the spinal cord, and is often referred to as the ‘second brain' due to its autonomous functions^1^. Organized into two types of ganglia, the myenteric and submucosal plexuses, the ENS is composed of motor neurons, sensory neurons and interneurons, and it is responsible for regulating vital functions such as gut motility and homeostasis^2^,^3^. Damage to the ENS is associated with inflammatory bowel disease, irritable bowel syndrome and other functional GI disorders^4^,^5^,^6^, which affect one quarter of the world's population and result in over 21.7 million hospitalizations and $141.8 billion in costs each year^7^,^8^. ENS dysfunction often accompanies systemic conditions such as obesity and diabetes, and emerging evidence suggests a link between Parkinson's disease and the ENS^9^,^10^,^11^.

Compared with the central nervous system, less is known about the ENS circuitry and its electrophysiological dynamics. For example, although ENS dysfunction is known to be associated with diseases like functional GI disorders, the exact electrophysiology is less clear^12^,^13^. As opposed to the brain and spinal cord, which can be imaged via cranial and spinal windows and recorded via implantable electrodes^14^,^15^,^16^, a major limitation in deciphering the ENS circuitry is our inability to optically image or electrically record ENS activities in live animals. Particular challenges for ENS recording include: the peristatic movements of the intestine, high background noise associated with surrounding longitudinal and circular muscles, lack of a fixed surface (for example, the skull or spine) to stabilize the device and maintain good contact, and the highly immunogenic gastrointestinal (GI) environment that interferes with ENS activities (in contrast, the central nervous system is much more immune tolerant towards foreign substrates)^17^,^18^,^19^. Gut movement also makes it extremely challenging to track the same groups of enteric neurons over multiple imaging sessions. Furthermore, given our limited knowledge about the ENS electrophysiology and high background noise, it is important to develop capabilities of simultaneous optical/electrical recording to take advantage of available transgenic reporter mice to validate and interpret recorded data.

The ENS has been imaged previously in an acute setup, where the intestine was brought out of the abdominal cavity, and the mouse was killed after the imaging session^20^. This method has several limitations if being applied to ENS recording: (i) the external environment and stretching the intestine out of the abdomen create artefacts due to ENS sensitivity to mechanical and chemical stimuli; (ii) the imaging duration is limited and (iii) this setup does not allow chronic monitoring over an extended period, which is important for understanding ENS-related physiology in health and disease.

An intravital abdominal window was previously developed to image intestinal epithelial stem cells^21^. In this setup, the intestine was glued to the coverslip on the window, which was also kept in place by glue. However, the glue causes substantial local inflammation, which could interfere with ENS activity, and makes this approach ill-suited for studying ENS. Other factors also make this approach impractical for chronic (repeated) studies of the ENS. The tendency of coverslip breakage hinders repeated imaging sessions and prevents integration of electrical recording devices. Peristaltic movements often cause the intestine to break off from the glue and detach from the window. Adhering the intestine to the window can cause intestinal obstruction or chronic inflammation, leading to high-mortality rates, fibrosis and mucus buildup, preventing repeated measures of the ENS function over the course of the experiment.

To overcome these limitations, a three-dimensional (3D)-printed insert is designed and surgically implanted into the animal to stabilize the intestine without blocking its motility functions. Through a customized abdominal intravital window, we manage to chronically image the same location in the mouse gut over several days and observe neuronal firing using the transgenic Pirt-GCaMP3 mice. By further integrating a transparent graphene sensor into the window, we demonstrate the ability to perform concurrent optical and electrical recording of ENS activity, with high spatiotemporal resolution to detect waveforms of single action potentials (APs) from individual enteric neurons. We use these novel capabilities to analyse the effects of various chemical and biomolecular stimuli and optogenetic manipulations on the gut, showing that such *in vivo*, real-time detection methods provide unique information necessary for the understanding of gut functionality *in vivo.*

## Results

### *In vivo* imaging using abdominal window

We designed a customized window to overcome the limitations found in earlier window designs. We started by surgically inserting a window into the fascia and skin layer of the mouse abdomen. The window uses thermally resistant, borosilicate glass, which is also much less susceptible to damage and less likely to break than conventional glass coverslips. Below the window, a 3D-printed insert holds the intestine in place while providing support and prevent ischaemia (Supplementary Fig. 1). Unlike a chemical adhesive, the implant is not adhered to the intestine, minimizing inflammation. Meanwhile, it can be tightened to reduce tissue movement without blocking proper digestion functions and peristalsis, extending the lifetime of the mouse. In other applications, such as intravital cranial and spinal windows, implants are placed at locations inaccessible to the mice, but abdominal windows are subject to constant scratching. We therefore developed a protocol using vet wrap to prevent the mouse from damaging the window, which substantially prolonged mouse survival and avoided inflammation (Supplementary Video 1). The mice implanted with the window consistently live up to 3 months, until they are killed for humane reasons.

**Figure 1:**
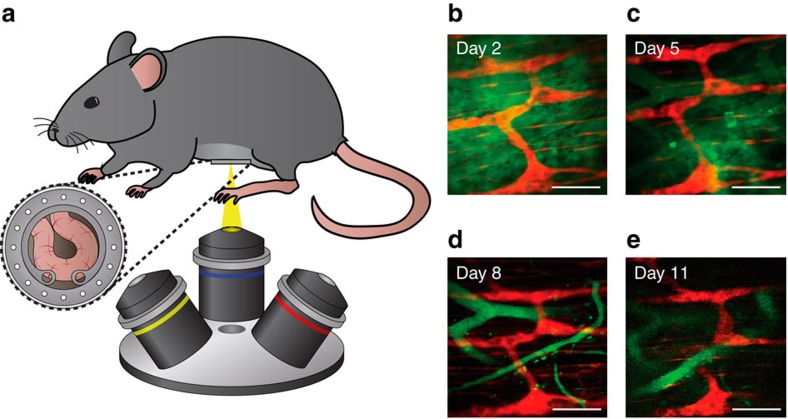
*In vivo* chronic multiphoton microscopy of ENS with abdominal window. (**a**) Schematic of *in vivo* multiphoton microscopy. (**b**–**e**) Days 2, 5, 8 and 11 after abdominal surgery, respectively, of *in vivo* multiphoton imaging in Wnt1-cre:tdTomato mice. The ENS is labelled with tdTomato (red), and the vasculature is labelled with FITC-dextran (green). Scale bar, 100 μm.

We demonstrated that it is possible to image the ENS *in vivo* using this setup. *Wnt1-cre:tdTomato* mice were chronically imaged with inverted multiphoton microscopy (Fig. 1a). In this transgenic strain, enteric neurons and other neural crest progeny are labelled with tdTomato^22^,^23^. The grid-like myenteric plexus formed by individual neurons is clearly visible through the transplanted abdominal window. To track the same set of enteric neurons on different days, fluorescein isothiocyanate (FITC)-dextran was injected retro-orbitally into the mouse to stain the vasculature, which served as a reference roadmap to locate the same region of the ENS (Fig. 1b–e and Supplementary Fig. 2). During subsequent imaging sessions, we were able to identify the same neurons using this vasculature roadmap. The plexus and vasculature morphology remained largely unchanged 11 days after the implantation surgery.

### Detection of ENS activity via graphene electrodes

To perform simultaneous electrical recording, we needed to integrate electrodes onto the abdominal window. The electrical recording device needs to be: (i) transparent to allow imaging through the device, (ii) inert and biocompatible to minimize fibrosis and inflammation, (iii) highly sensitive to detect ENS APs from background noise and (iv) durable for long-term chronic monitoring. To meet these stringent requirements, we developed a graphene-based electrode array that can be integrated onto the abdominal window to measure electrical activities in the ENS. Consisting of a carbon sheet one atom thick, graphene offers unique advantages as a transparent material with superb conductivity (zero bandgap), biocompatibility (an inert surface) and strength (Young's modulus higher than diamond and 207 times stronger than steel by weight)^24^,^25^.

The chip contains nine graphene electrodes, ranging from 50 μm by 50 μm to 500 μm by 500 μm. Graphene was grown on a copper substrate via a vertical cold-wall chemical vapour deposition system and was transferred onto a transparent quartz surface. Gold interconnects, 150 nm thick, were evaporated onto the surface and protected by insulating photoresist that left the contact pads exposed (Supplementary Fig. 3). To integrate the graphene sensor onto the abdominal window for surgical implantation, a piece of borosilicate glass, containing eight via-holes, 250 μm diameter, was inserted into a titanium ring and fixed in place by a horseshoe spring. The graphene chip was then secured to the borosilicate glass using transparent, insulating epoxy. Wires were threaded through the glass and secured to the connection pads using silver epoxy. The glass via-holes and connection pads were sealed using transparent insulating epoxy to protect the connection (Supplementary Fig. 4).

**Figure 2:**
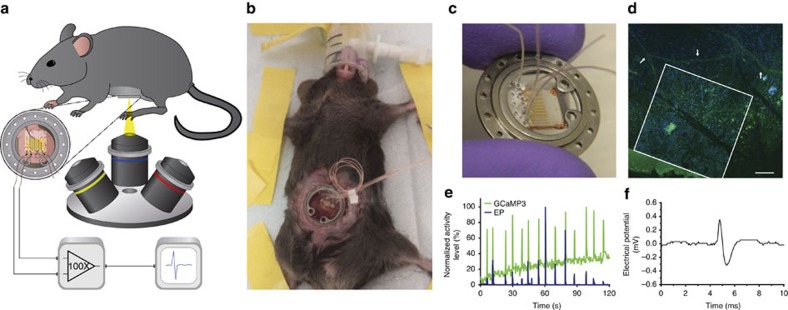
Simultaneous optical/electrical recording *in vivo* using integrated graphene sensor and abdominal window. (**a**) Schematic of *in vivo* optical/electrical recording. The graphene sensor is in close proximity to the small intestine, within the titanium ring of the surgical implant. (**b**) Mouse with integrated graphene sensor and abdominal window, several days after abdominal window implantation surgery. (**c**) The implantable abdominal window integrated with the graphene sensor. (**d**) Graphene electrode implanted in *Pirt-GCaMP3* mouse. One graphene electrode (500 μm by 500 μm, white box) in contact with the surrounding nerves (green, identified with white arrows) and surrounding collagen tissue (blue). Scale bar, 100 μm. (**e**) Simultaneous multiphoton microscopy and electrical potential (EP) recording *in vivo*, in *Pirt-GCaMP3* mouse. GCaMP3 fluorescence (green) and electrical potential (blue) are plotted, with absolute value of each signal normalized to 100%. (**f**) A high-temporal resolution waveform of an *in vivo* recorded AP.

The integration of graphene sensor and abdominal window provides us with the capability of simultaneous optical imaging and electrical recording in live mice. We surgically implanted the device into *Pirt-GCaMP3* mice such that the customized 3D insert held the small intestine in contact with the exposed graphene electrodes without disrupting digestive and motility functions (Fig. 2a–d). Through the transparent graphene sensor, the inverted multiphoton microscope was able to track activation of individual neurons in the myenteric plexus, which were fluorescently labelled via their genetically encoded GCaMP3 indicator for Ca^2+^ ion flux^26^ (Supplementary Fig. 5 and Supplementary Video 2).

We then performed simultaneous optical and electrical recording to track activated neurons in the myenteric plexus of *Pirt-GCaMP3* mice. The timing of multiphoton microscopy and the electrical recording apparatus are synchronized by a timing gate. Bursts of activity in GCaMP3 fluorescence (measured by multiphoton microscopy) and in electrical potential (measured by the graphene sensor) maintained high synchrony *in vivo* (Fig. 2e). The synchrony between electrical recording and optical imaging validates the mutual dependence between electrical potential spikes and the GCaMP fluorescence associated with APs. The combined optical/electrical measurements confirmed that our integrated abdominal device was indeed capable of capturing ENS APs, which is important given our limited knowledge about the ENS and the noise from the surrounding muscle layers. Compared with optical imaging, the sensitive graphene sensor provides much higher temporal resolution with greater signal-to-noise ratio, which allowed us to examine the detailed biphasic waveforms of ENS APs (Fig. 2f).

### ENS activity in response to chemical stimulations

**Figure 3:**
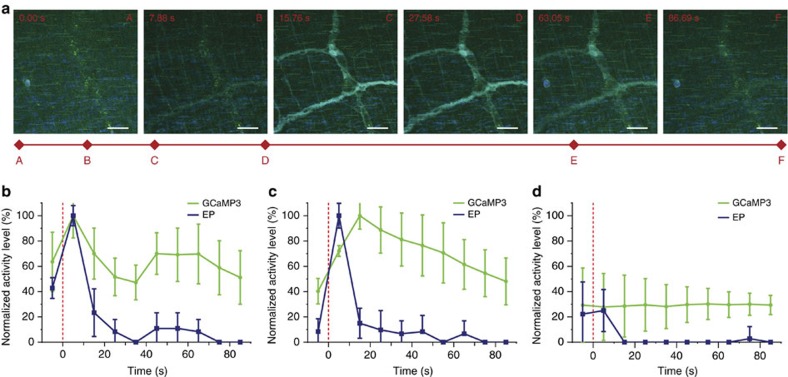
Unpaired analysis of fluorescent and electrical potential response to chemical stimuli. (**a**) Frames from time-lapse recording of GCaMP3-expressing nerves during serotonin stimulation. The activated nerves fluoresce (green) due to stimulation, before fading back to low fluorescence levels. Scale bar, 50 μm. (**b**–**d**) Average GCaMP3 fluorescence (green) and average percentage of electrical recording (blue), after (**b**) acetylcholine, (**c**) serotonin or (**d**) PBS stimulations plotted relative to the time of stimulation (*n*=3 and 5 for GCaMP3 and electrical potential respectively; error bars show standard deviation (s.d.)). Stimulus was applied at time 0 (red dashed line).

ENS activities are spatiotemporally regulated by various neurotransmitters. For instance, the majority of the serotonin in the human body is produced in the GI tract to regulate intestinal movements^27^,^28^. However, there was previously no means of studying real-time ENS response to these neurotransmitters *in vivo*. As proof-of-concept, we used the integrated device to study ENS response to acetylcholine and serotonin. Repeated optical imaging and electrical recording in distinct animals demonstrated that GCaMP3 fluorescence and electrical potential consistently followed the same trend upon delivery of acetylcholine or serotonin, consistent with activation of enteric neurons (Fig. 3a–d). The electrical potential measured by the graphene sensor showed a faster deactivation rate than GCaMP3 fluorescence, highlighting the relative slow response kinetics of GCaMP relative to electrical potential. Our data suggest that optical imaging with GCaMP3 technology provides better spatial resolution for tracking AP location, whereas electrical measurement provides higher temporal resolution to track AP dynamics. The integrated device combines both to provide a more comprehensive picture of ENS spatiotemporal activities in real time in live animals.

There are a range of Food and Drug Administration-approved small-molecule drugs that act via acetylcholine and serotonin receptors. Bethanechol targets muscarinic acetylcholine receptors, which can increase GI muscular tone and urinary retention^29^,^30^. Tegaserod is an agonist for the serotonin receptor 5-HT_4_ to stimulate GI motility and peristatic reflexes, which could relieve bloating and constipation associated with irritable bowel syndrome^31^. However, acetylcholine and serotonin also activate other receptors that the drugs do not target (for example, nicotinic acetylcholine receptors).

**Figure 4:**
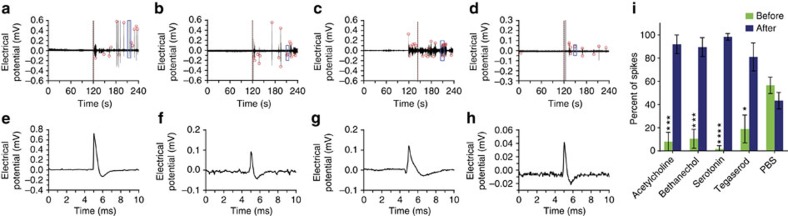
Electrical response to chemical stimuli *in vivo*. (**a**–**d**) Neural response to *in vivo* stimulation by (**a**) acetylcholine, (**b**) bethanechol, (**c**) serotonin and (**d**) tegaserod. Stimulus occurs at 120 s (red dashed line), and the electrical potential (black) and spikes (red circles) are plotted over time. (**e**–**h**) Waveforms of representative APs from above recordings (blue box) are shown at higher temporal resolution. (**i**) The effect of chemical stimulation is compared between acetylcholine, bethanechol, serotonin, tegaserod and a PBS control group. The average percentage of spikes that occur before stimulation (green) and the average percentage that occur after (blue) are plotted for each group (*n*=4; error bars show s.d.). All repeats consisted of recordings lasting 240 s, with stimulation at 120 s. Measurements of individual recordings used to calculate the average percentages are shown in Supplementary Fig. 7. *P<0.05; ***P<0.005; ****P<0.001, one sided *t*-test.

We compared ENS responses with acetylcholine, bethanechol, serotonin and tegaserod, which were delivered via intraperitoneal (IP) injections. Experiments were designed to record 2 min of unperturbed activity before the chemical stimulation and an additional 2 min to observe post-stimulus activity. Compared with the phosphate-buffered saline control, both native transmitters and the drugs were capable of increasing activity in the ENS (Fig. 4a–d). The graphene sensor captured detailed waveforms of the activated APs (Fig. 4e–h). Quantification of repeated recordings in different mice confirmed an increase in the number of spikes when stimulated by either neurotransmitter or agonist drug (Fig. 4i, Supplementary Figs 6,7 and Supplementary Table 1). Chronic recordings of the same ENS regions over 5 days showed that activities of enteric neurons were either consistently responsive or non-responsive to the serotonin stimuli (Supplementary Fig. 8). Application of tetrodotoxin (TTX), which blocks voltage-gated sodium channels, consistently suppressed measured ENS activities *in vivo* (Supplementary Fig. 9). 200 ml of 133 μM Tegaserod was also delivered via oral gavage, which was sufficient to stimulate ENS activities (Supplementary Fig. 10).

### ENS activity in response to 470 nm light stimulations

**Figure 5:**
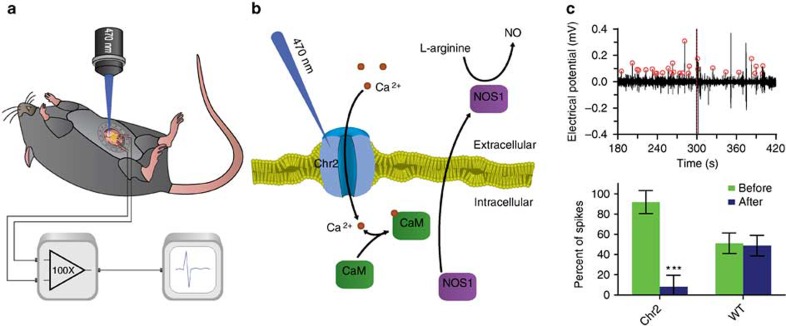
Optogenetic application of integrated graphene sensor and abdominal window. (**a**) A schematic for *in vivo* stimulation of *Nos1-creERT2:Chr2* mice with 470 nm light. (**b**) Mechanistic schematic for 470 nm inhibition in Nos1-creERT2:Chr2 mice. (**c**) Representative *in vivo* recording with 470 nm stimulation in Chr2-expressing mice. 470 nm light stimulation occurs at 300 s (red dashed line). Electrical potential (black) is plotted over time. (**d**) The effect of *in vivo* 470 nm light stimulation is compared between *Nos1-creERT2:Chr2* mice (Chr2) and wild-type mice (WT). The average percentage of spikes that occur before stimulation (green), and the average percentage that occur after (blue) are plotted for each group (*n*=4; error bars show s.d.). Only data 120 s before and after stimulation are analysed during repeats. ***P<0.005, one sided *t*-test.

The transparent integrated device also enables us to take advantage of optogenetic technology. In optogenetic mice, light can be applied through the abdominal window and the transparent graphene sensor to stimulate light-gated ion channels, and subsequent ENS responses will be recorded by the graphene sensor. For proof-of-principle, the device was surgically implanted in *Nos1-creER*^*T2*^*:Chr2* mice. A 470-nm light stimulus was applied onto the small intestine through the abdominal window and transparent graphene sensor (Fig. 5a). In these mice, incident 470 nm light opens genetically modified Ca^2+^ channels, activating neuronal nitric oxide synthase (NOS)-positive GABAergic neurons, which produce nitric oxide that acts as a neurotransmitter to affect neurons, the interstitial cells of Cajal and muscle (Fig. 5b)^32^,^33^.

To demonstrate 470 nm light inhibition in the nitrergic neurons in the *NOS1-creER*^*T2*^*:Chr2* animals, recordings were designed to have 5 min of unperturbed activity before a 15-s 470 nm light stimulation, followed by an additional 5 min of recording to observe post-stimulus activity. We found that stimulation at 470 nm in Chr2-expressing mice reduced spontaneous activity in naturally active regions of the ENS by 83.66% (Fig. 5c,d, Supplementary Fig. 11 and Supplementary Table 2). In contrast, the same 470-nm incident light stimulation had little effect in wild-type mice, which do not express the genetically modified Ca^2+^ channels. Repeated 470 nm light stimulation ∼6 min apart in chronically implanted *Nos1-creER*^*T2*^*:Chr2* mice yielded a decrease in inhibition efficiency, as activities had not fully recovered from the previous stimulation (Supplementary Fig. 12). It should be noted that as NOS1-expressing neurons also commonly co-express other neurotransmitters, such as vasoactive intestinal peptide (VIP), further studies are needed to interpret the physiological basis of these results. Nevertheless, the experiments provide proof of principle for the ability to detect change in ENS activity by targeting specific cell type.

Collectively, we demonstrated simultaneous optical/electrical recording of the ENS in real time by developing an integrated abdominal implant device. The recording can be repeated in live animals carrying this device. Characterization of the ENS response to neurotransmitter/chemical stimuli and optogenetic manipulation was demonstrated. The device has the potential to aid our understanding of the ENS, its role in diseases and its interaction with infection, inflammation, microbiota and the gut–brain axis.

## Methods

### Ethics statement

All animal procedures were reviewed and approved by the Cornell University Institutional Care and Use Committee (protocol no. 2010-0100 and 2015-0029) and were conducted in strict accordance with the recommendations in the Guide for the Care and Use of Laboratory Animals, published by the National Institutes of Health.

### Animal models

Pirt-GCaMP3 mice were generated previously^26^. The *Wnt1-cre:tdTomato* mice were generated by crossing *Tg(Wnt1-cre)11Rth Tg(Wnt1-GAL4)11Rth/J* (Jackson Laboratory: 003829) with *Ai14* (Jackson Laboratory: 007914). The *Nos1-creER*^*T2*^*:Chr2* mice were generated by crossing *B6:129S-Nos1*^*tm1.1(cre/ERT2)Zjh*^*/J* (Jackson Laboratory: 014541) with *Ai32* (Jackson Laboratory: 012569). Gene expression was induced in *Nos1-creER*^*T2*^*:Chr2* mice by tamoxifen IP injection at 75 mg kg^−1^ daily for 5 days.

### Graphene sensor manufacturing

Graphene was grown in a chemical vapour deposition system on copper substrate and transferred using poly(methyl methacrylate) to a quartz substrate^34^. The chip was then patterned with interconnects and a photoresist layer, using standard fabrication techniques at the Cornell NanoScale Science and Technology Facility. Gold interconnects are protected with a layer of photoresist, and they connect exposed contact pads to the exposed graphene electrodes (Supplementary Fig. 3a–d). The electrode array (Supplementary Fig. 3e) consists of nine graphene electrodes, ranging from 50 μm by 50 μm to 500 μm by 500 μm.

### Graphene sensor and window integration

Eight via-holes (250 μm diameter) were cut through the borosilicate glass (10 mm diameter) using a laser (VersaLaser VLS3.50). The graphene device was mounted on the glass with transparent epoxy (Hardman Epoxy, 04004). Stranded copper wire (Cooner Wire, CZ1101-A) carry the signal off chip and through via-holes in the window. Copper wire was fixed to gold contact pads using conductive epoxy (MG Chemicals, 83302-21G). Finally, transparent epoxy was used to protect and insulate the copper–gold interface. The transparent epoxy was also used to seal the copper wire in via-holes to maintain separation between the internal and external environments during surgical implantation (Supplementary Fig. 4).

### Recording from the graphene sensor

The electrical potential was recorded by two identical graphene electrodes of the array. From the copper wires, the signal was delivered to a differential AC amplifier (A-M Systems, 1,700) at a 20-kHz sampling rate, where it received a × 100 gain and band-pass filter between 300 Hz and 5 kHz. Following amplification, the signal was processed using data acquisition board (NI, BNC-2110) and software (NI, Signal Express).

### Chronic abdominal window surgery

We used adult mice ranging from 6 to 12 weeks of age. Animals were anaesthetized with isoflurane (4% vol/vol induction with 2–3% maintenance) and eye ointment was applied. The abdomen was shaved and cleaned with povidone iodine and ethanol (70% (vol/vol) in water). After a local injection of bupivacaine (0.1% (wt/vol) in saline, 0.1 ml per mouse) and atropine sulfate (0.05 mg kg^−1^), a circular incision (7 mm) was made into the skin over the mouse abdomen followed by a circular incision (4 mm radius) into the muscle wall, which was prepared inside of skin incision. During all procedures when the mouse was under anaesthesia, body temperature was maintained at 37.5 °C by a thermostatically regulated heating pad, and 0.1 ml of glucose (5% (wt/vol) in physiological saline) was applied every hour. A sterile, 3D-printed insert was implanted into the surgical site. A portion of the intestine was moved on top of the insert. The insert was fixed in place with sutures attached to the abdominal muscle. The abdominal window was placed over the incision site, and the ring was attached to the surrounding skin by using instant adhesive (Loctite 406). For surgical procedures involving the sensor, the graphene chip had been adhered to the secured coverslip before the procedure. Once the adhesive had been secured, a 20-mm band of self-adhering vet wrap (3 M VetRap) was wrapped around the abdomen to prevent damage to the surgical site while the mouse is awake and to protect the wire connections. After the surgery, the animal was removed from anaesthesia and allowed to recover (Supplementary Video 1). Subcutaneous injections of dexamethasone sodium phosphate (0.2 mg kg^−1^; American Regent, Inc.) and ketoprofen (5 mg kg^−1^; Fort Dodge) were given every 24 h for 3 days following surgery.

### *In vivo* multiphoton imaging

*In vivo* imaging was conducted on a Zeiss LSM880 confocal/multiphoton inverted microscope. Fluorescence emission was collected in two different channels: 505–545 nm wavelengths for FITC and green fluorescent protein detection, and 560–650 nm for tdTomato detection. Green fluorescent protein excitation wavelength was 900 nm, and simultaneous FITC and tdTomato excitation wavelengths were 1,000 nm. Excitation was performed by an InSight DeepSee laser. A × 10/0.5 air EC Plan-Neofluar objective was used to visualize the cells *in vivo*. Mice were anaesthetized with isofluorane (4% (vol/vol) with 2–3% maintenance), and eye ointment was applied. Vet wrap was removed to expose the glass window, and the mice were placed on a 3D-printed imaging stage. To visualize the vasculature, 50 μl of FITC-Dextran dye (Sigma-Aldrich) was injected retro-orbitally. After the imaging was completed, mouse abdomens were rewrapped in fresh vet wrap to prevent scratching. Imaging was performed once every other day to allow the mouse time to recover between imaging sessions.

### Chronic chemical and 470 nm light stimulations

Post-surgery, mice were anaesthetized with isofluorane (4% vol/vol with 2–3% maintenance), and eye ointment was applied. Vet wrap was removed to expose the graphene sensor and copper wires (Supplementary Video 3). The copper wires were connected to the amplifier. Chemical stimulation consisted of IP injection of either 100 μM acetylcholine (Sigma-Aldrich), 100 μM bethanechol (VWR), 10 μM serotonin (Fisher), 133 μM tegaserod (Santa Cruz Biotech), 1 μM Tetrodotoxin citrate (Abcam) or saline (Fisher), in 50 μl quantities. TTX was applied to three mice in a terminal procedure. For the oral drug treatment, mice were given 200 μl of 133 μM tegaserod (Santa Cruz Biotech) via oral gavage. Mice were not exposed to more than four chemical stimulations in a single procedure and stimulation procedures were done at least 2 days apart to allow the mouse to recover. Light stimulations were executed using an AURA light engine at 230 mW. Stimulation consisted of 470 nm light for 15 s through the abdominal window and graphene electrodes. Electrical recording consisted of 2 min of background activity, stimulation, followed by at least 2 min of post-stimulus activity. Oral gavage required a 3-min delay between the pre-stimulus and post-stimulus recording to allow for proper tegaserod administration via oral gavage. After the imaging was completed, mouse abdomens were rewrapped in fresh vet wrap to prevent irritation. Only one electrical recording session was performed every 2 days to allow the mouse to recover between procedures.

### Statistics

Healthy mice, between 6 and 12 weeks of age, were selected at random for experiments. Each recording session (replicate) was conducted in a new animal each time, except for chronic recording. Recordings were repeated in four different animals for each chemical stimulation (acetylcholine, bethanechol, serotonin or tegaserod), with a total of 20 recording sessions in distinct animals. Recordings with TTX application were repeated in three different animals. Chronic recordings were compared between responsive and non-responsive animals recorded on days 1, 3 and 5. 470 nm stimulation experiments were repeated in four *NOS1-creER*^*T2*^*:Chr2* mice and four wild-type mice. *P*-values were determined using paired, one-sided *t*-tests.

### Data analysis

Electrical data were analysed in MATLAB to identify spikes. For consistency between recordings, a lower threshold was determined using a minimum signal-to-noise ratio. An upper threshold was also used to filter out artefacts in the signal caused by violent perturbations because of stimulation. The code was not changed between analyses of experiments to maintain consistency and prevent bias.

Once spikes had been identified, the effect of each stimulant was determined by calculating the percentage of spikes occurring before and after the stimulus in each recording. For consistency, the time window being analysed before the stimulus is the same length of time being analysed after the stimulus. This time window is at least 2 min for all recordings.

Optical data were analysed in ImageJ software. Mean grey value was calculated for regions containing dynamic GCaMP fluorescence. The mean grey value, proportional to the amount of fluorescence, was used to determine the relative activity level during the optical recording.

### Data availability

The data that support the findings of this study are available from the corresponding author upon request.

## Additional information

**How to cite this article:** Rakhilin, N. *et al*. Simultaneous optical and electrical *in vivo* analysis of the enteric nervous system. *Nat. Commun.* 7:11800 doi: 10.1038/ncomms11800 (2016).

## Supplementary Material

Supplementary Information Supplementary Figures 1-12 and Supplementary Tables 1-2

Supplementary Movie 1 Healthy, active, Pirt-GCaMP3 mouse freely moving around cage three days after surgical implantation of the integrated device.

Supplementary Movie 2 Time lapse recording of GCaMP fluorescence (green) due to serotonin stimulation

Supplementary Movie 3 Pirt-GCaMP3 mouse, three days after surgery, being prepared for electrical recording

Peer Review File

## References

[b1] Gershon M. D. The enteric nervous system: a second brain. Hosp Pract (1995) 34, 31–32 35-38, 41-32 passim (1999). 10418549 10.3810/hp.1999.07.153

[b2] Campbell I. Gut motility and its control. Anaesth. Intensive Care Med. 13, 59–61 (2012).

[b3] Matteoli G. & Boeckxstaens G. E. The vagal innervation of the gut and immune homeostasis. Gut 62, 1214–1222 (2013). 23023166 10.1136/gutjnl-2012-302550 PMC3711371

[b4] Bouche P., Le Forestier N., Maisonobe T., Fournier E. & Willer J. C. Electrophysiological diagnosis of motor neuron disease and pure motor neuropathy. J. Neurol. 246, 520–525 (1999). 10463350 10.1007/s004150050397

[b5] Jones M. P., Dilley J. B., Drossman D. & Crowell M. D. Brain-gut connections in functional GI disorders: anatomic and physiologic relationships. Neurogastroenterol. Motil. 18, 91–103 (2006). 16420287 10.1111/j.1365-2982.2005.00730.x

[b6] Grundy D. . Fundamentals of neurogastroenterology: basic science. Gastroenterology 130, 1391–1411 (2006). 16678554 10.1053/j.gastro.2005.11.060

[b7] Gershon M. D. & Tack J. The serotonin signaling system: from basic understanding to drug development for functional GI disorders. Gastroenterology 132, 397–414 (2007). 17241888 10.1053/j.gastro.2006.11.002

[b8] Digestive Diseases Statistics for the United States Report No. 13-3873 (National Digestive Diseases Information Clearinghouse, National Institutes of Health, Bethesda, MD, (2013).

[b9] Mayer E. A. Gut feelings: the emerging biology of gut-brain communication. Nat. Rev. Neurosci. 12, 453–466 (2011). 21750565 10.1038/nrn3071 PMC3845678

[b10] Chandrasekharan B. & Srinivasan S. Diabetes and the enteric nervous system. Neurogastroenterol. Motil. 19, 951–960 (2007). 17971027 10.1111/j.1365-2982.2007.01023.x PMC3711013

[b11] Phillips R. J., Walter G. C., Wilder S. L., Baronowsky E. A. & Powley T. L. Alpha-synuclein-immunopositive myenteric neurons and vagal preganglionic terminals: autonomic pathway implicated in Parkinson's disease? Neuroscience 153, 733–750 (2008). 18407422 10.1016/j.neuroscience.2008.02.074 PMC2605676

[b12] Chey W. D., Kurlander J. & Eswaran S. Irritable bowel syndrome: a clinical review. JAMA 313, 949–958 (2015). 25734736 10.1001/jama.2015.0954

[b13] Rogler G. Where are we heading to in pharmacological IBD therapy? Pharmacol Res. 100, 220–227 (2015). 26277232 10.1016/j.phrs.2015.07.005

[b14] Canales A. . Multifunctional fibers for simultaneous optical, electrical and chemical interrogation of neural circuits *in vivo*. Nature Biotechnol. 33, 277–284 (2015). 25599177 10.1038/nbt.3093

[b15] Cianchetti F. A., Kim D. H., Dimiduk S., Nishimura N. & Schaffer C. B. Stimulus-evoked calcium transients in somatosensory cortex are temporarily inhibited by a nearby microhemorrhage. PLoS ONE 8, e65663 (2013). 23724147 10.1371/journal.pone.0065663 PMC3665593

[b16] Farrar M. J. . Chronic *in vivo* imaging in the mouse spinal cord using an implanted chamber. Nat. Methods 9, 297–302 (2012). 22266542 10.1038/nmeth.1856 PMC3429123

[b17] Warden M. R., Cardin J. A. & Deisseroth K. Optical neural interfaces. Annu. Rev. Biomed. Eng. 16, 103–129 (2014). 25014785 10.1146/annurev-bioeng-071813-104733 PMC4163158

[b18] Carson M. J., Doose J. M., Melchior B., Schmid C. D. & Ploix C. C. CNS immune privilege: hiding in plain sight. Immunol. Rev. 213, 48–65 (2006). 16972896 10.1111/j.1600-065X.2006.00441.x PMC2633103

[b19] Khodagholy D. . Highly conformable conducting polymer electrodes for in vivo recordings. Adv. Mater. 23, H268–H272 (2011). 21826747 10.1002/adma.201102378

[b20] Goto K. . *In vivo* imaging of enteric neurogenesis in the deep tissue of mouse small intestine. PLoS ONE 8, e54814 (2013). 23382976 10.1371/journal.pone.0054814 PMC3561410

[b21] Ritsma L. . Intestinal crypt homeostasis revealed at single-stem-cell level by *in vivo* live imaging. Nature 507, 362–365 (2014). 24531760 10.1038/nature12972 PMC3964820

[b22] Becker L., Kulkarni S., Tiwari G., Micci M. A. & Pasricha P. J. Divergent fate and origin of neurosphere-like bodies from different layers of the gut. Am. J. Physiol. Gastrointest. Liver Physiol. 302, G958–G965 (2012). 22361728 10.1152/ajpgi.00511.2011 PMC3362075

[b23] Hao M. M. . Early emergence of neural activity in the developing mouse enteric nervous system. J. Neurosci. 31, 15352–15361 (2011). 22031881 10.1523/JNEUROSCI.3053-11.2011 PMC6703522

[b24] Stankovich S. . Graphene-based composite materials. Nature 442, 282–286 (2006). 16855586 10.1038/nature04969

[b25] Yang K., Feng L., Shi X. & Liu Z. Nano-graphene in biomedicine: theranostic applications. Chem. Soc. Rev. 42, 530–547 (2013). 23059655 10.1039/c2cs35342c

[b26] Kim Y. S. . Central terminal sensitization of TRPV1 by descending serotonergic facilitation modulates chronic pain. Neuron 81, 873–887 (2014). 24462040 10.1016/j.neuron.2013.12.011 PMC3943838

[b27] Berger M., Gray J. A. & Roth B. L. The expanded biology of serotonin. Annu. Rev. Med. 60, 355–366 (2009). 19630576 10.1146/annurev.med.60.042307.110802 PMC5864293

[b28] Kendig D. M. & Grider J. R. Serotonin and colonic motility. Neurogastroenterol. Motil. 27, 899–905 (2015). 26095115 10.1111/nmo.12617 PMC4477275

[b29] Bryant B. D. & Knights K. M. Pharmacology for Health Professionals 3rd edn Elsiever Australia (2011).

[b30] Abrams P. . Muscarinic receptors: their distribution and function in body systems, and the implications for treating overactive bladder. Br. J. Pharmacol. 148, 565–578 (2006). 16751797 10.1038/sj.bjp.0706780 PMC1751864

[b31] Camilleri M. Review article: tegaserod. Aliment Pharmacol. Ther. 15, 277–289 (2001). 11207504 10.1046/j.1365-2036.2001.00925.x

[b32] Bornstein J. C., Marks K. A., Foong J. P., Gwynne R. M. & Wang Z. H. Nitric oxide enhances inhibitory synaptic transmission and neuronal excitability in Guinea-pig submucous plexus. Front. Neurosci. 4, 30 (2010). 20589236 10.3389/fnins.2010.00030 PMC2904599

[b33] Grider J. R. & Murthy K. S. Autoinhibition of endothelial nitric oxide synthase (eNOS) in gut smooth muscle by nitric oxide. Regul. Pept. 151, 75–79 (2008). 18926858 10.1016/j.regpep.2008.09.005 PMC4864723

[b34] Muñoz N. Design, Fabrication And Geometric Optimization Of Graphene Electrodes For Electrochemical Detection Cornell University (2014).

