# Supplementary material for: Simultaneous optical and electrical in vivo analysis of the enteric nervous system
Source: Nat Commun. 2016 Jun 7;7:11800. doi: 10.1038/ncomms11800 (PMC4899629; doi:10.1038/ncomms11800)
Supplement: Supplementary Information — Supplementary Figures 1-12 and Supplementary Tables 1-2 [file ncomms11800-s1.pdf]

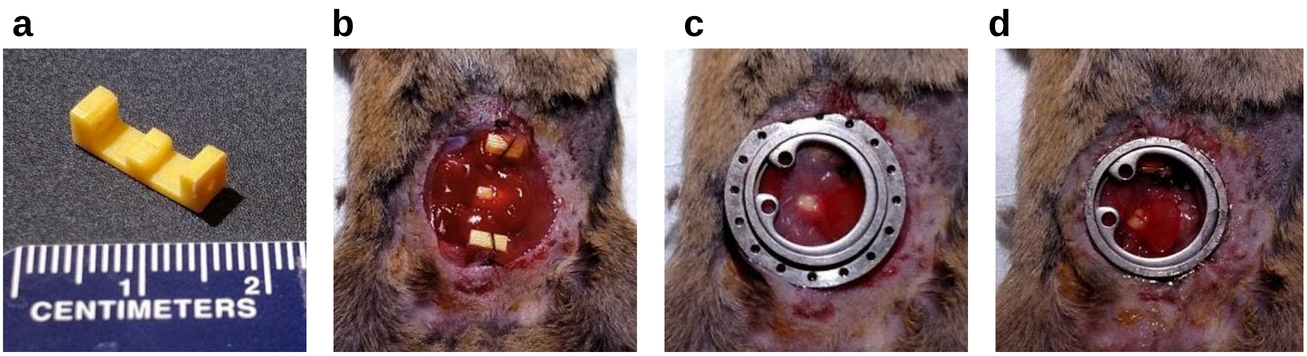

Supplementary Figure 1. Mouse surgery using 3D printed stabilizing insert.

(**a**) 3D printed insert designed to fix intestine in place and prevent ischemia. (**b**) 3D printed insert implanted underneath the intestine of an anesthetized mouse with sutures attaching the insert to the abdominal muscle layer. (**c**) Titanium window placed over the surgical opening with the insert visible through the window. (**d**) Titanium window is permanently adhered to the skin.

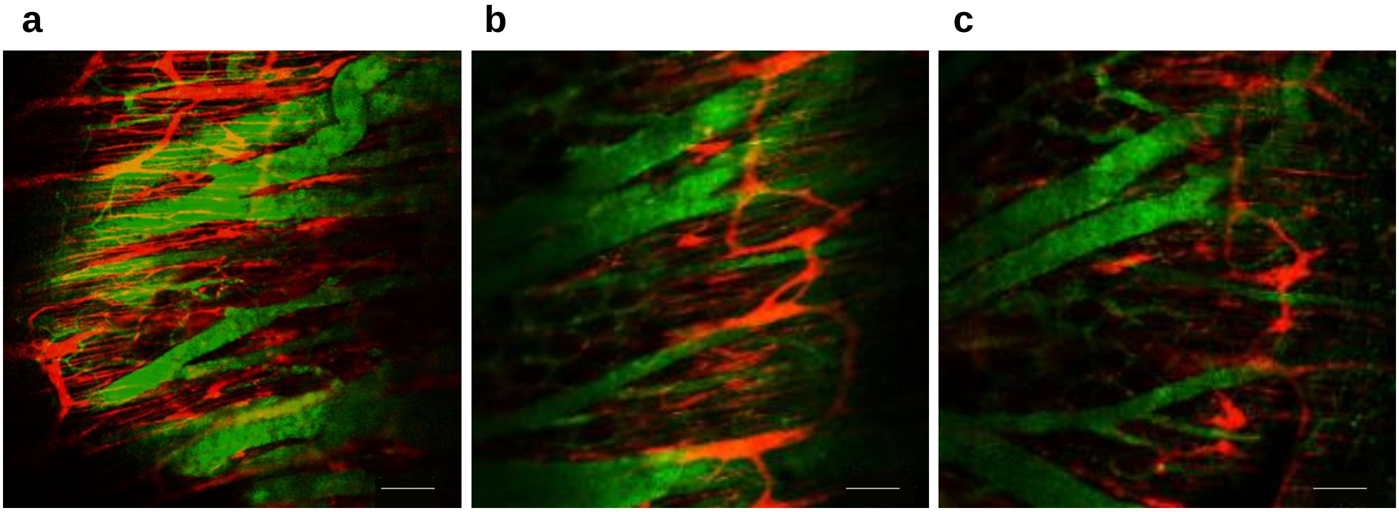

Supplementary Figure 2. Wnt1-cre:tdTomato mouse chronic study.

(**a**-**c**) Images of the ENS from 5, 7 and 9 days after surgery, respectively. Mice were implanted with the abdominal window on day 0. The same location in the vasculature (green) and the same set of nerves (red) are shown for each day. Scale bar, 100 μm.

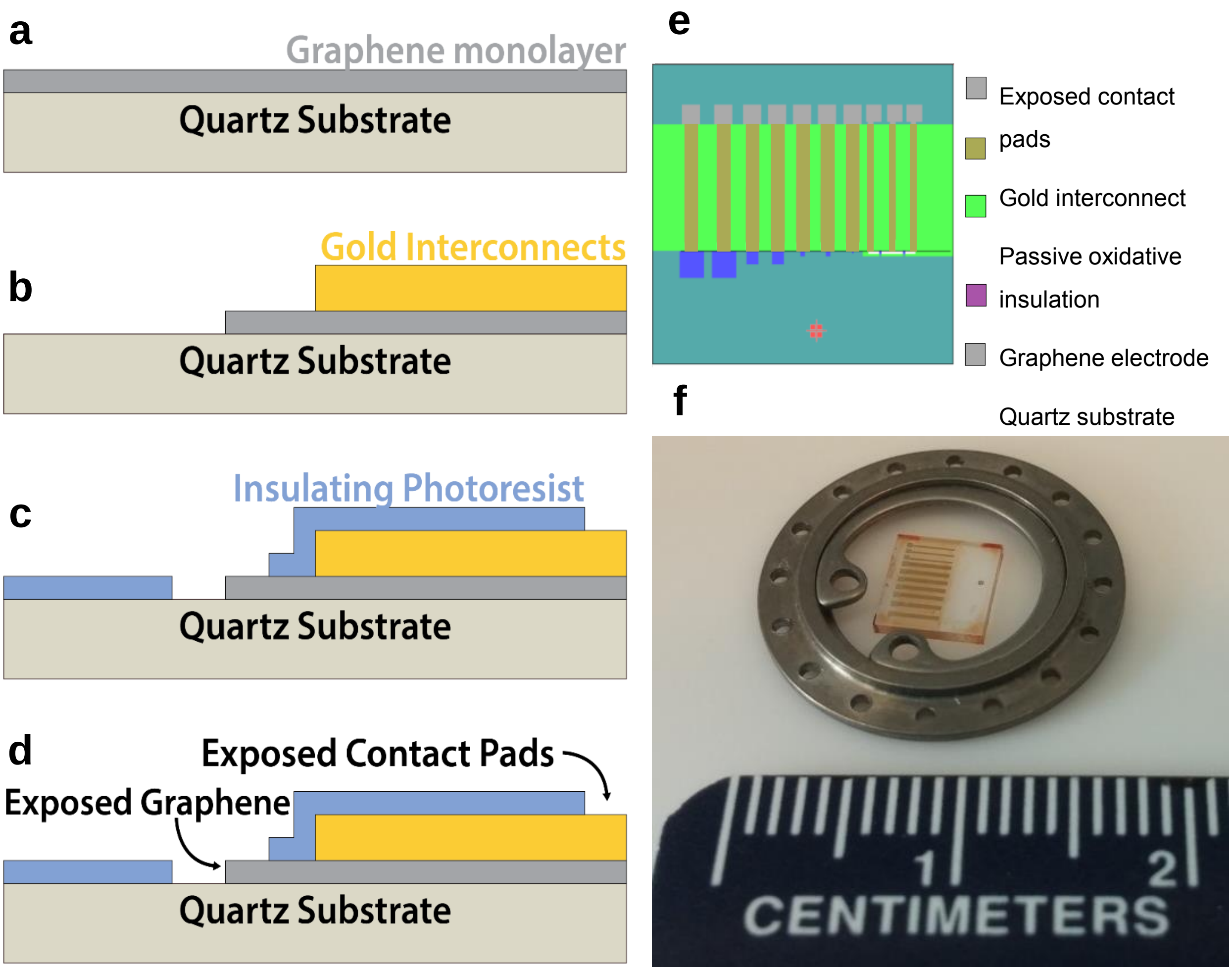

Supplementary Figure 3. Graphene electrode fabrication.

(**a**) Graphene is transferred onto a transparent quartz surface from a copper substrate. (**b**) Gold is evaporated onto the graphene layer to serve as interconnects (150 nm thick). (**c**) Photoresist is applied to preserve the gold interconnects. (**d**) The graphene and gold contact pads remain exposed. (**e**) Schematic of the chip layers and organization. (**f**) The graphene chip inside the titanium ring.

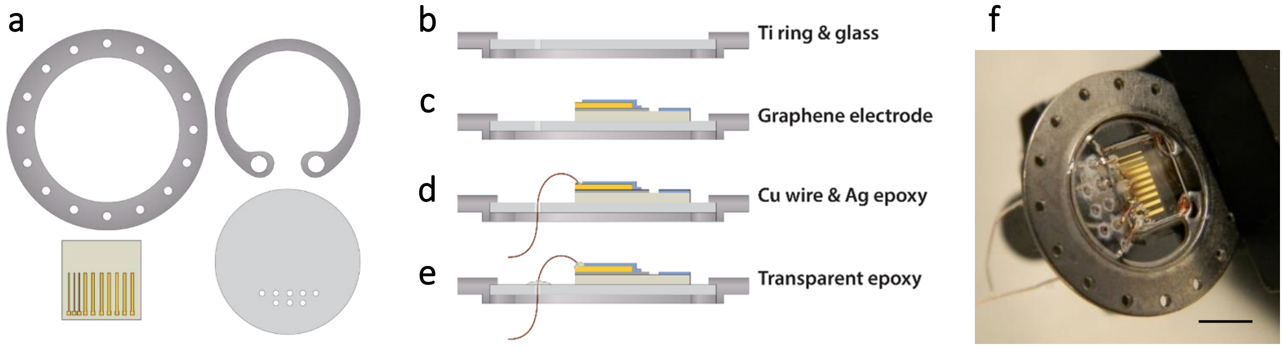

Supplementary Figure 4. Graphene electrode integration with abdominal window.

(**a**) The titanium ring, horseshoe spring, borosilicate glass containing eight via holes (250 μm diameter), and graphene sensor. (**b**) The borosilicate glass is inserted into the titanium ring and fixed in place by the horseshoe spring. (**c**) The chip is secured to the borosilicate glass using transparent, insulating epoxy. (**d**) Wires are threaded through the glass and secured to the connection pads using conductive, silver epoxy. (**e**) Connection pads are sealed using transparent, insulating epoxy, to protect the connection. (**f**) The integrated graphene sensor and abdominal window. Scale bar, 5 mm.

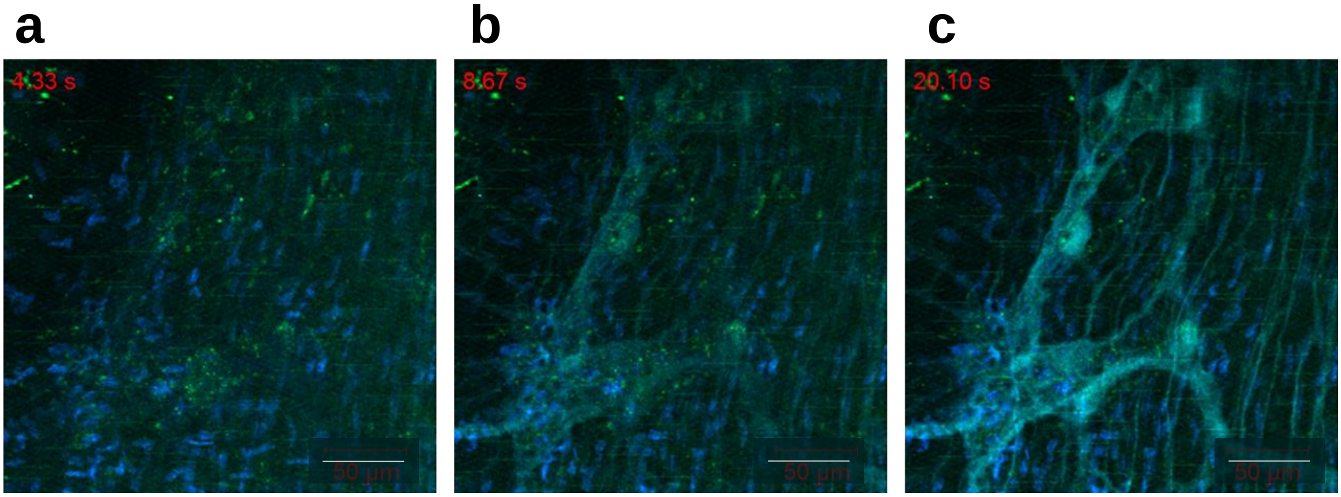

Supplementary Figure 5. Time lapse recording of GCaMP3 nerves stimulated with serotonin.

(a) Initial level of GCaMP3 fluorescence, without stimulus. (**b**) GCaMP3 fluorescence activates in nerves (green) during serotonin stimulation. (**c**) Nerves show a high level of fluorescence after serotonin stimulation. Scale bar, 50 µm.

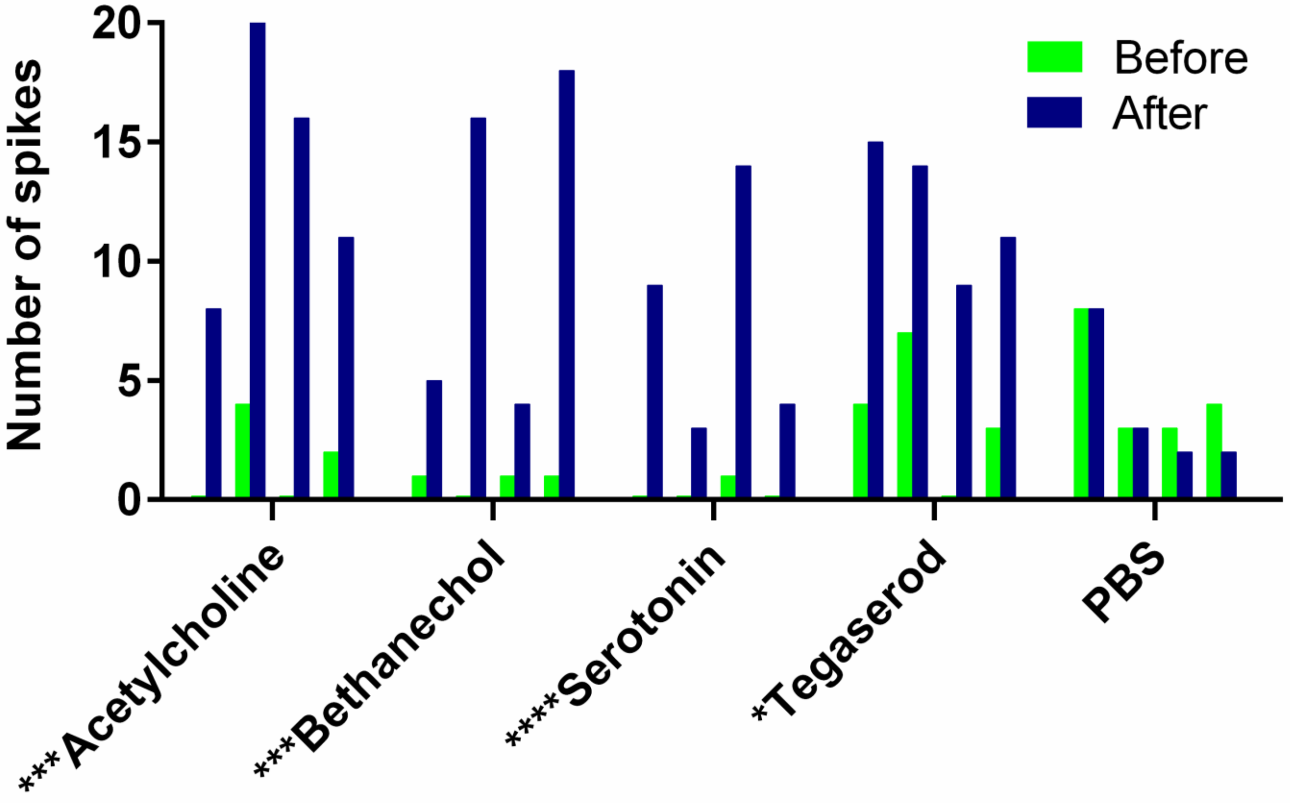

Supplementary Figure 6. Spike count before and after stimulation.

Number of spikes before (green) and after (blue) stimulation from each recording with acetylcholine, bethanechol, serotonin, tegaserod, or PBS stimuli. Each pair of green and blue bars represents the measured number of spikes before and after stimulation from the same recording session.

a

b

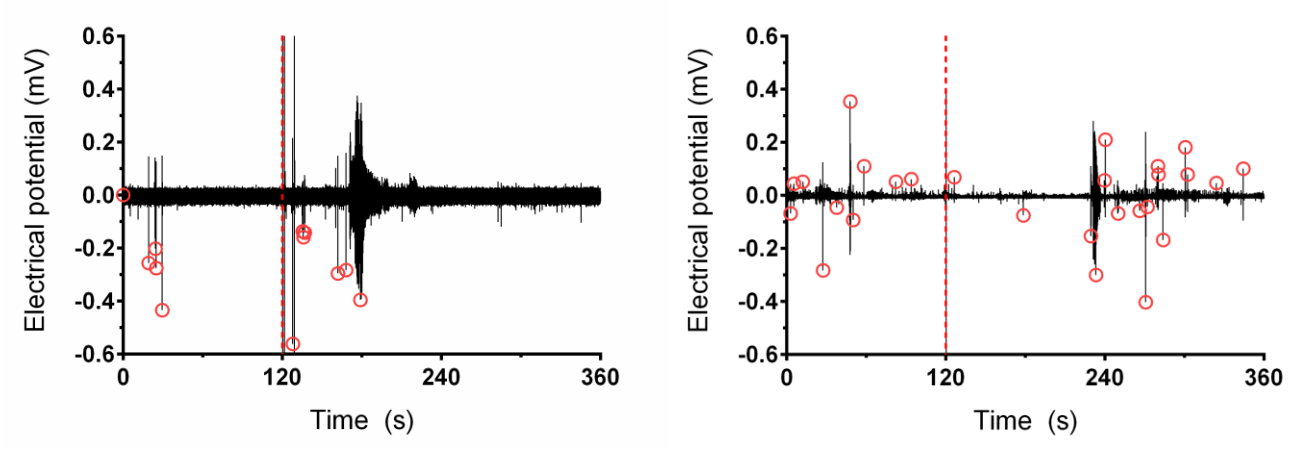

Supplementary Figure 7. Extended recordings of enteric neuron stimulation.

Neural response to *in vivo* stimulation by (**a**) bethanechol and (**b**) tegaserod beyond 2 minutes after stimulation. Stimulus occurs at 120 seconds.

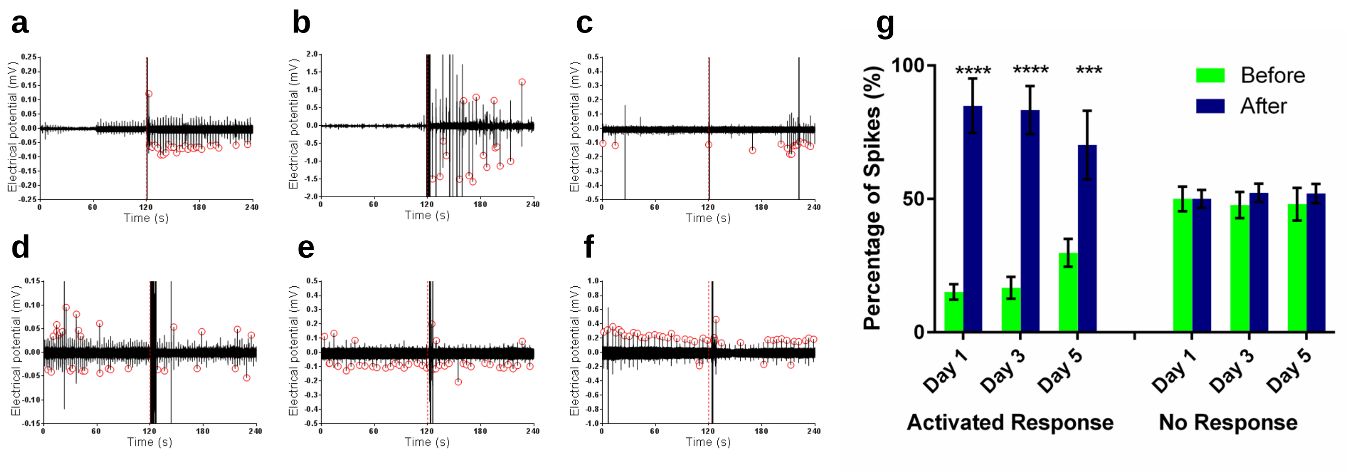

Supplementary Figure 8. Chronic *in vivo* recordings over 5 days.

(**a**-**c**) Representative activated responses to serotonin on day 1, 3 and 5, respectively. (**d**-**f**) Representative limited or no responses to serotonin on day 1, 3 and 5, respectively. (**g**) Neural activity before (green) and after (blue) serotonin stimulation over 5 days with cases of activated response and no response (n=5; error bars show s.d.); p-value determined by one sided t-test.

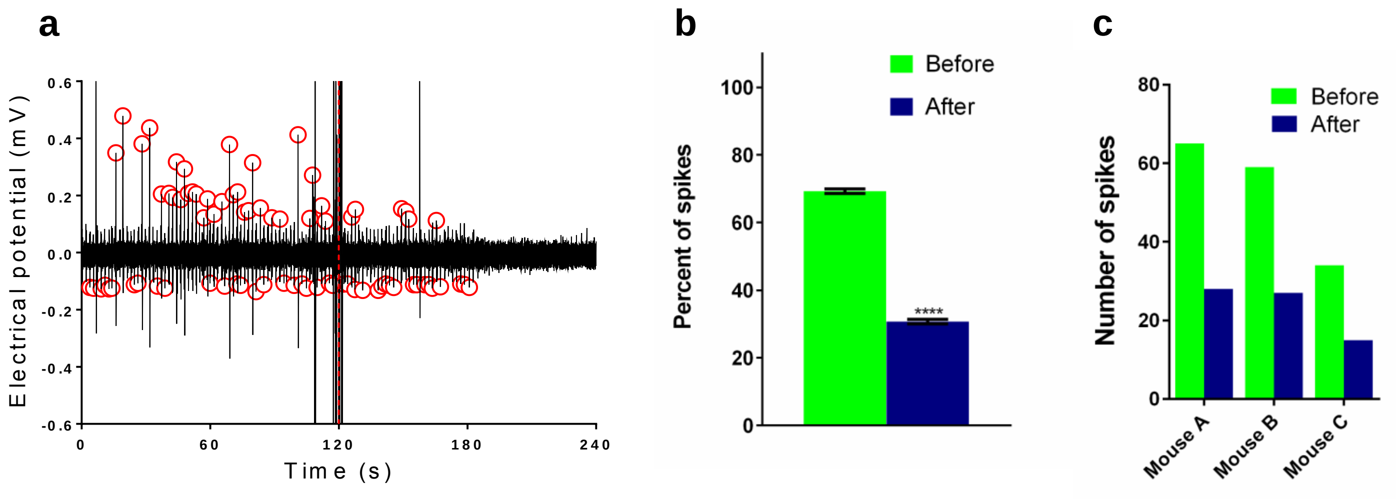

Supplementary Figure 9. Enteric neuron inhibition with tetrodotoxin.

Neuron firing was inhibited through treatment with 1mM tetrodotoxin (TTX). (**a**) TTX application occurs at 120 seconds (red dashed line), and the electrical potential and spikes are plotted against time. (**b**) The average percentage of spikes that occur before stimulation (green), and the average percentage that occur after (blue) are shown (n=3; error bars show s.d.). One sided t-test was used to determine the p-value. (**c**) Number of spikes before (green) and after (blue) TTX stimulation in distinct mice.

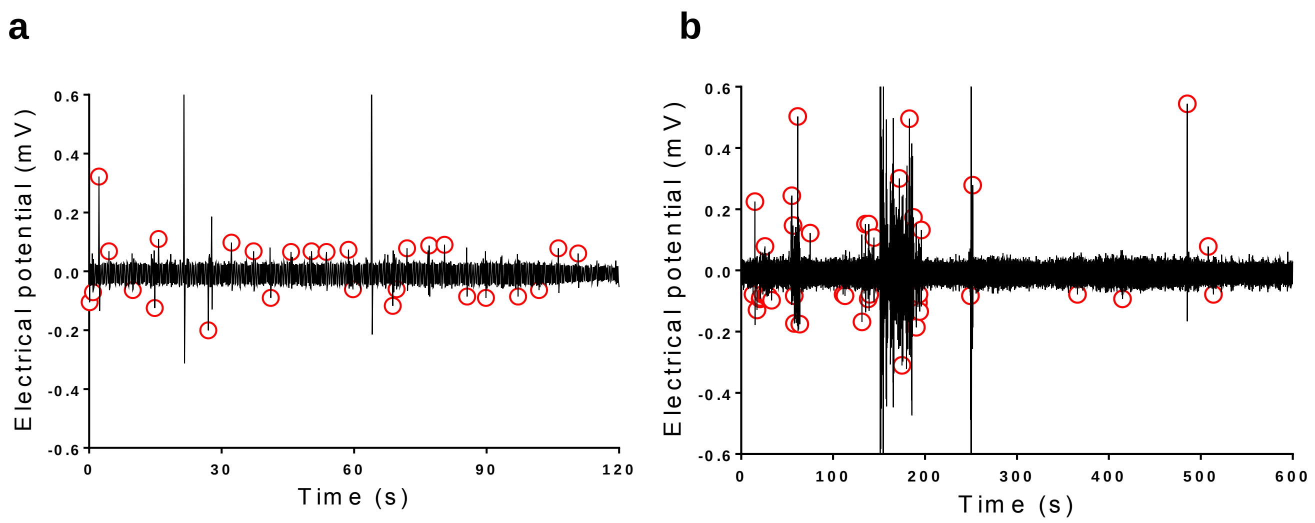

Supplementary Figure 10. Oral gavage of Tegaserod.

Mouse was treated with 200mL of 133 µM tegaserod via oral gavage, with enteric electrical potentials recorded before (**a**) and after (**b**) treatment. There is a 3 minute delay between the two recordings for gavage.

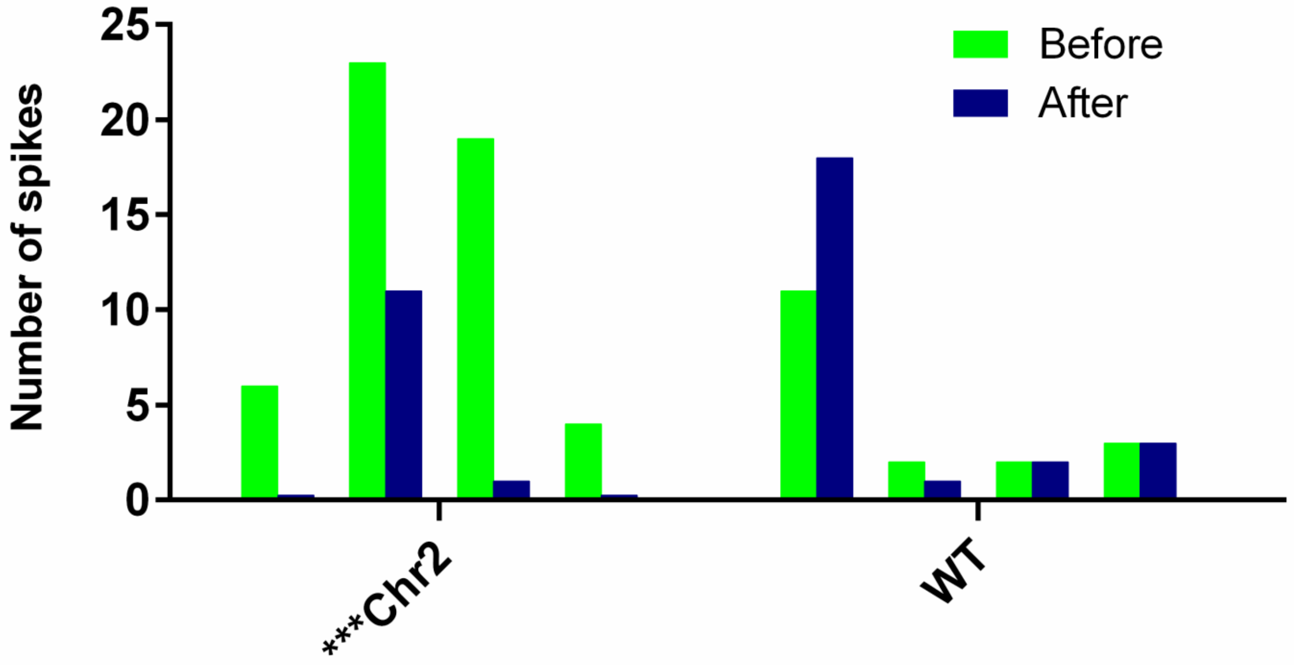

Supplementary Figure 11. Spike count before and after 470 nm stimulation.

Number of spikes before (green) and after (blue) 470 nm stimulation in Chr2-expressing mice and WT mice. Each pair of green and blue bars represents the measured number of spikes before and after stimulation from the same recording session.

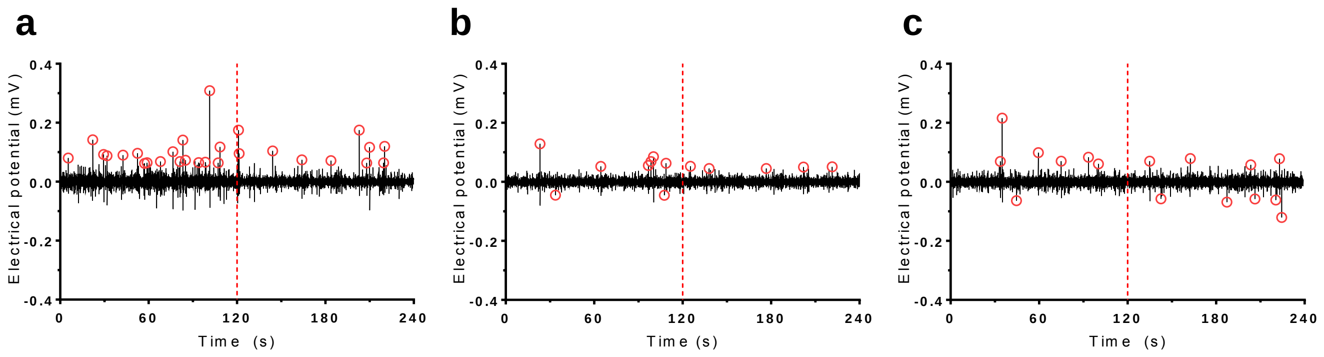

Supplementary Figure 12. Repeated 470 nm stimulation of Chr2-expressing mice.

(**a-c**) Stimulation of Nos1-creERT2:Chr2 mice with 470 nm light while recording electrical potential. Consecutive stimulations, lasting 15 seconds each, approximately 6 minutes apart (10:54:00, 11:01:25, 11:06:42) show a decrease in the effectivity of NOS1 inhibition.

Supplementary Table 1: Data summary for chemical stimulation.

Stimulant	Acetylcholine	Bethanechol	Serotonin	Tegaserod	PBS
Pre-stimulus	8.01%	10.48%	1.67%	18.95%	56.57%
Post-stimulus	91.99%	89.52%	98.33%	81.05%	43.33%
Standard dev.	8.03%	8.15%	2.89%	12.01%	7.07%

Supplementary Table 1. Data summary for chemical stimulation.

Change in spikes due to chemical stimulant application in vivo. Recordings lasted for 240 seconds, and all stimulations occurred at 120 seconds. Acetylcholine, bethanechol, serotonin, and tegaserod had a significant increase in spikes (n=4, p<0.005, 0.005, 0.001 and 0.05 respectively), while the addition of PBS did not have a significant change in spikes (n=4, p>0.1).

Supplementary Table 2: Data summary for 470 nm light stimulation.

**Mouse strain**	**Nos1-creER^{T2}:Chr2**	**C57BL/6J (WT)**
Pre-stimulus	91.83%	51.15%
Post-stimulus	8.17%	48.85%
Standard dev.	11.45%	10.22%

Supplementary Table 2. Data summary for 470 nm light stimulation.

Change in spikes due to light stimulation in vivo. Nos1-creERT2:Chr2 mice and C57BL/6J mice (WT) were used as experimental and control groups respectively. Recordings lasted 240 seconds, and the light stimulus was applied at 120 seconds, for a duration of 15 seconds. The Nos1-creERT2:Chr2 mice showed a significant decrease in spikes (n=4, p<0.005) after 470 nm light stimulation, while the control mice showed no significant change in activity (n=4, p>0.1).
