# Supplementary material for: Simultaneous optical and electrical in vivo analysis of the enteric nervous system
Source: Nat Commun. 2016 Jun 7;7:11800. doi: 10.1038/ncomms11800 (PMC4899629; doi:10.1038/ncomms11800)
Supplement: Peer Review File [file ncomms11800-s5.pdf]

Editorial Note: this manuscript has been previously reviewed at another journal that is not operating a transparent peer review scheme. This document only contains reviewer comments and rebuttal letters for versions considered at Nature Communications.

REVIEWERS' COMMENTS:

Reviewer #1 (Remarks to the Author):

The authors have addressed the questions that arose in the prior review.

Reviewer #2 (Remarks to the Author):

Rakhilin et al have addressed the majority of the previous issues in a satisfactory manner. Using the 3D printed intestinal insert has improved their stabilization significantly, and addition of some requested images and control experiments has strengthened their manuscript. We commend the authors on a nice novel method for imaging neuronal activity in the gastrointestinal tract.

Reviewer #3 (Remarks to the Author):

The authors reasonably revised the original version of the manuscript according to my suggestions.

RESPONSE TO REVIEWERS:

We thank the reviewers for their enthusiasm. They did not raise any further points that need to be addressed.
